# Author Correction: Ontogeny and transcriptional regulation of Thetis cells

**DOI:** 10.1038/s41586-026-10770-7

**Published:** 2026-06-16

**Authors:** Yoselin A. Paucar Iza, Tyler Park, Eliyambuya Baker, Gayathri Shibu, Tilman Hoelting, Greyson Feather, Anushka Yadav, Yollanda Franco Parisotto, Zihan Zhao, Blossom Akagbosu, Marc Elosua Bayes, Logan Fisher, Lucas M. James, Jianping Ma, Benjamin D. Philpot, Behdad Afzali, Christina Leslie, Chrysothemis C. Brown

**Affiliations:** 1 Howard Hughes Medical Institute, New York, NY USA; 2 Immunology and Microbial Pathogenesis Program, Weill Cornell Medicine Graduate School of Medical Sciences, New York, NY USA; 3 Immuno-Oncology Program, Memorial Sloan Kettering Cancer Center, New York, NY USA; 4 Computational and Systems Biology Program, Memorial Sloan Kettering Cancer Center, New York, NY USA; 5 Department of Cell Biology and Physiology, University of North Carolina at Chapel Hill, Chapel Hill, NC USA; 6 Neuroscience Center, University of North Carolina at Chapel Hill, Chapel Hill, NC USA; 7 Carolina Institute for Developmental Disabilities, University of North Carolina at Chapel Hill, Chapel Hill, NC USA; 8 Immunoregulation Section, Kidney Diseases Branch, National Institute of Diabetes and Digestive and Kidney Diseases, NIH, Bethesda, MD USA; 9 Department of Pediatrics, Memorial Sloan Kettering Cancer Center, New York, NY USA; 10 Present Address: Boston Children’s Hospital, Harvard Medical School, Boston, MA USA

**Keywords:** Haematopoiesis, Innate immune cells, Mucosal immunology

Correction to: *Nature* 10.1038/s41586-026-10198-z Published online 3 February 2026

Following publication of this article, we were alerted via PubPeer to an inadvertent duplication error of values in Extended Data Fig. 6c. During transcription of the data into GraphPad Prism, the data values for TCP (pLN 11 weeks) were inadvertently duplicated from the LTi cell column of the same block, and for Spleen P12, the first two rows of data values for DC1 and DC2 were inadvertently duplicated from the corresponding rows of the pLN P12 block. In addition, column headings for Spleen P12 were also found to be transposed (TCP, TC I-IV instead of TC I-IV, TCP).

Prompted by this comment, we carried out a careful review of all other figures and their Source Data. In Fig. 5i, the Source Data for LTo cells at day 13 inadvertently included values from a replicate experiment that was not part of this analysis. The figure panel and Source Data have been replaced with the correct values.

Separately, during this review we identified several errors that were confined to the Source Data files and did not affect the published figures, which were generated directly from the raw data. In the Source Data for Fig. 3d, one row of TLP values was inadvertently transcribed twice from the raw data. In the Source Data for Extended Data Figs. 3, 7d and 9e, column headings were transposed. In the Source Data for Extended Data Fig. 7j, the values transcribed corresponded to the RORγt MFI rather than the FLT3 MFI. In each case the Source Data files have been corrected; the figures and all analyses are unaffected.

Figure 5, Extended Data Fig. 6 and associated Source Data have been updated in the HTML and PDF versions of this Article. For transparency, the original and corrected figures are available as Supplementary Information accompanying this amendment.

These corrections do not alter the overall pattern, biological interpretation or conclusions of the study. The authors thank the reader for bringing these errors to our attention and apologize for any confusion.

**Supplementary information** is available in the online version of this amendment.

## Supplementary information

Original and corrected Fig. 5i, Extended Data Fig. 6c.

