# Supplementary material for: Ontogeny and transcriptional regulation of Thetis cells
Source: Nature. 2026 Feb 3;653(8114):538–47. doi: 10.1038/s41586-026-10198-z (PMC13171621; doi:10.1038/s41586-026-10198-z)
Supplement: Supplementary file 1 — Supplementary Figs. 1 and 2. [file 41586_2026_10198_MOESM1_ESM.pdf]

nature portfolio

https://doi.org/10.1038/s41586-026-10198-z

Supplementary information

Ontogeny and transcriptional regulation of Thetis cells

In the format provided by the authors and unedited

Ontogeny and transcriptional regulation of Thetis cells

Authors: Yoselin A. Paucar Iza[1,2], Tyler Park[3], Eliyambuya Baker[1], Gayathri Shibu[1,2], Tilman Hoelting[1], Greyson Feather[1], Anushka Yadav[1], Yollanda Franco Parisotto[1], Zihan Zhao[1], Blossom Akagbosu[1], Marc Elosua Bayes[1+], Logan Fisher[1,2], Lucas M. James[4], Jianping Ma[5], Benjamin D. Philpot[4], Behdad Afzali[5], Christina Leslie[3], Chrysothemis C. Brown[1,2,6]

Affiliations:

[1]Howard Hughes Medical Institute and Immuno-Oncology Program, Memorial Sloan Kettering Cancer Center, New York, USA

[2]Immunology and Microbial Pathogenesis Program, Weill Cornell Medicine Graduate School of Medical Sciences, New York, NY, USA

[3]Computational and Systems Biology Program, Memorial Sloan Kettering Cancer Center, New York, NY, USA

[4]Department of Cell Biology and Physiology, Neuroscience Center, and Carolina Institute for Developmental Disabilities, University of North Carolina at Chapel Hill, Chapel Hill, NC, USA.

[5]Immunoregulation Section, Kidney Diseases Branch, National Institute of Diabetes and Digestive and Kidney Diseases, NIH, Bethesda, Maryland, USA.

[6]Department of Pediatrics, Memorial Sloan Kettering Cancer Center, New York, NY, USA

+ Current Affiliation: Boston Children's Hospital, Harvard Medical School, Boston, MA, USA

Supplementary Fig. 1. FACS-isolation of IL7R-fate mapped and RORγt(Venus)$^{+}$ cells

Supplementary Fig. 2 FACS-isolation of TCs for Smart-seq3

Other Supplementary Material for this manuscript includes the following:

Supplementary Table 1. Metadata for samples processed for Flex scRNA-seq on IL7R-fate mapped and RORγt^{+} cells.

Supplementary Table 2. List of differentially expressed genes for TCP, LTiP and TLP clusters from Fig. 1I.

Supplementary Table 3. List of antibodies used for flow cytometry.

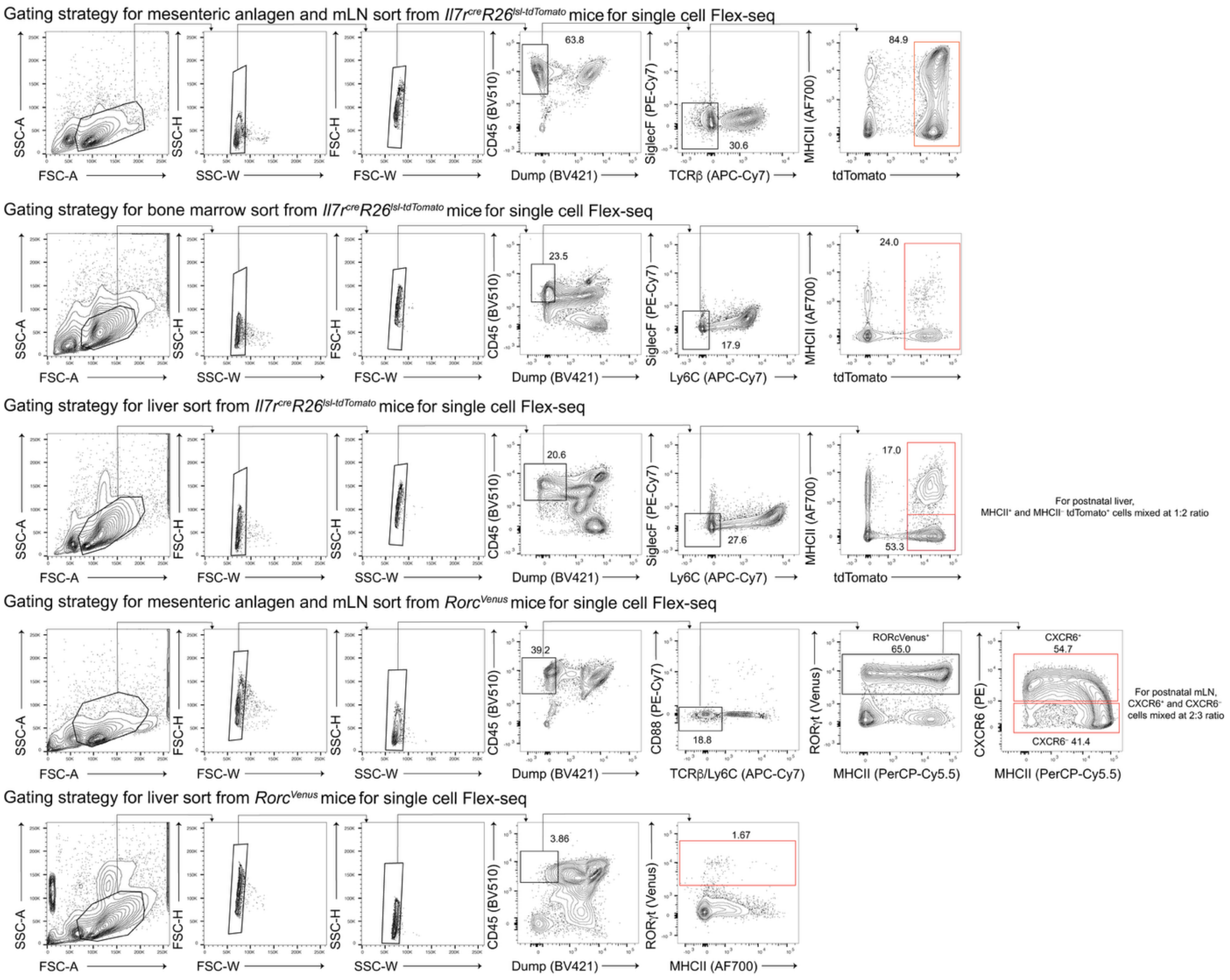

Supplementary Fig. 1. FACS-isolation of IL7R-fate mapped and RORγt(Venus)$^+$ cells

FACS isolation of CD45$^+$Lin$^-$ IL7R-fate mapped and Lin$^-$RORγt(Venus)$^+$ cells in E17.5–18.5 fetal liver, bone marrow (BM) and mesenteric anlagen, and P7 liver, BM and mesenteric lymph nodes (mLN). Lineage markers for each tissue and strain provided in Supplementary Table 1.

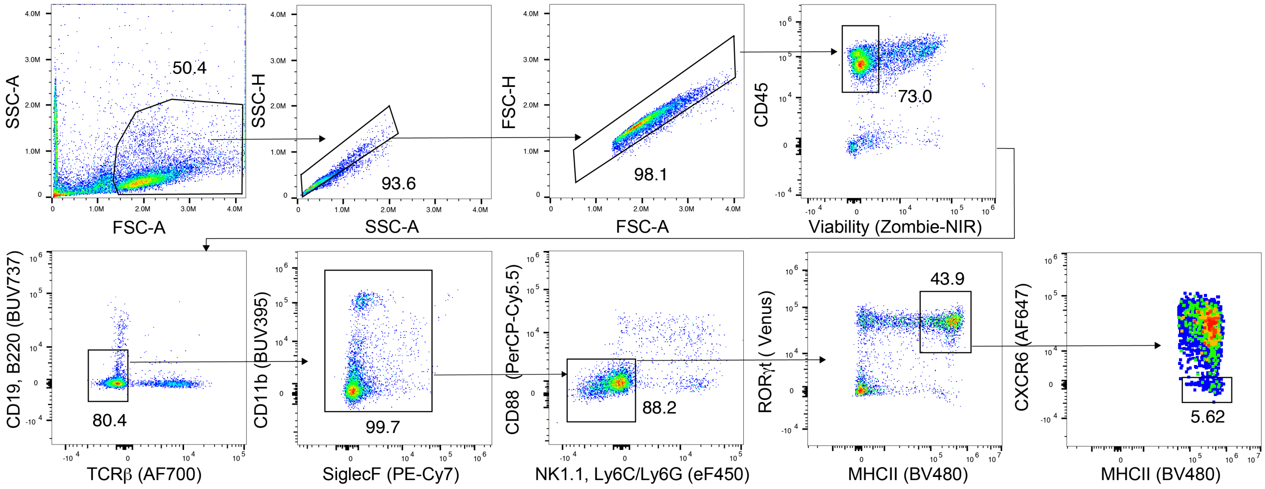

Supplementary Fig. 2 FACS-isolation of TCs for Smart-seq3

FACS isolation of TCs from mLN of 2-week-old *Rorc*^{Venus} mice (pooled from 14 biological replicates) for Smart-seq 3 (SS3) analysis.
