# Supplementary material for: Ontogeny and transcriptional regulation of Thetis cells
Source: Nature. 2026 Feb 3;653(8114):538–47. doi: 10.1038/s41586-026-10198-z (PMC13171621; doi:10.1038/s41586-026-10198-z)
Supplement: Supplementary file 2 — Reporting Summary [file 41586_2026_10198_MOESM2_ESM.pdf]

nature portfolio

Corresponding author(s): Chrysothemis Brown

Last updated by author(s): January 12, 2025

Reporting Summary

Nature Portfolio wishes to improve the reproducibility of the work that we publish. This form provides structure for consistency and transparency in reporting. For further information on Nature Portfolio policies, see our Editorial Policies and the Editorial Policy Checklist.

Statistics

For all statistical analyses, confirm that the following items are present in the figure legend, table legend, main text, or Methods section.

n/a	Confirmed	
☐	☒	The exact sample size (*n*) for each experimental group/condition, given as a discrete number and unit of measurement
☐	☒	A statement on whether measurements were taken from distinct samples or whether the same sample was measured repeatedly
☐	☒	The statistical test(s) used AND whether they are one- or two-sided<br>*Only common tests should be described solely by name; describe more complex techniques in the Methods section.*
☒	☐	A description of all covariates tested
☐	☒	A description of any assumptions or corrections, such as tests of normality and adjustment for multiple comparisons
☐	☒	A full description of the statistical parameters including central tendency (e.g. means) or other basic estimates (e.g. regression coefficient) AND variation (e.g. standard deviation) or associated estimates of uncertainty (e.g. confidence intervals)
☐	☒	For null hypothesis testing, the test statistic (e.g. *F*, *t*, *r*) with confidence intervals, effect sizes, degrees of freedom and *P* value noted<br>*Give P values as exact values whenever suitable.*
☒	☐	For Bayesian analysis, information on the choice of priors and Markov chain Monte Carlo settings
☒	☐	For hierarchical and complex designs, identification of the appropriate level for tests and full reporting of outcomes
☒	☐	Estimates of effect sizes (e.g. Cohen's *d*, Pearson's *r*), indicating how they were calculated

Our web collection on statistics for biologists contains articles on many of the points above.

Software and code

Policy information about availability of computer code

Data collection	Cytek Aurora v3.3.0 and Facs Diva v8.0.1 (BD Biosciences) for flow cytometry. 10X Chromium for single cell RNA-sequencing. Details for RNA-seq are included in the methods.
Data analysis	Flowjo v10.10.0 for flow cytometry data. GraphPad Prism v9 and v10 for statistical analyses.<br><br>All computational methods are detailed in the Methods. No custom code was created for this study. Scripts used to analyze scRNA-seq data are available at Github (https://github.com/pty0111/TC-progenitor-2026). The following softwares were used:<br>HARP v0.1.1, Cell Ranger v8.0.0, Seurat v4.4.0, SCimilarity v0.3.0, Celltypist v1.5.3, zUMIs v2.9.7e, STAR v2.7.11a, CellRank 2 v2.0.6, ArchR v1.0.3, chromVAR v1.14.0

For manuscripts utilizing custom algorithms or software that are central to the research but not yet described in published literature, software must be made available to editors and reviewers. We strongly encourage code deposition in a community repository (e.g. GitHub). See the Nature Portfolio guidelines for submitting code & software for further information.

Data

Policy information about availability of data

All manuscripts must include a data availability statement. This statement should provide the following information, where applicable:

- Accession codes, unique identifiers, or web links for publicly available datasets
- A description of any restrictions on data availability
- For clinical datasets or third party data, please ensure that the statement adheres to our policy

The mouse sequencing data is available through the Gene Expression Omnibus (accession number GSE316677) Sequencing data for the Human Gut Atlas has been published previously and the processed data is publicly available from https://www.gutcellatlas.org. Previously published scRNA/ATAC-seq data from Cabric et al. (GSE174405) and Akagbosu et al. (GSE294005) were referenced.

Human research participants

Policy information about studies involving human research participants and Sex and Gender in Research.

Reporting on sex and gender	N/A
Population characteristics	N/A
Recruitment	N/A
Ethics oversight	N/A

Note that full information on the approval of the study protocol must also be provided in the manuscript.

Field-specific reporting

Please select the one below that is the best fit for your research. If you are not sure, read the appropriate sections before making your selection.

☒ Life sciences ☐ Behavioural & social sciences ☐ Ecological, evolutionary & environmental sciences

For a reference copy of the document with all sections, see nature.com/documents/nr-reporting-summary-flat.pdf

Life sciences study design

All studies must disclose on these points even when the disclosure is negative.

Sample size	Sample size was determined based on preliminary data, and previously published data for similar experiments (PMID: 38677292, PMID: 31358996 and 40373113). In all experiments, a minimum of three mice per group were used, Details as to the number of replicates and sample size are included in the Methods or Figure Legends.
Data exclusions	Samples with insufficient cell numbers were excluded from the analyses (Figure 3c; <90 CD45.2+Lin–MHCII+).
Replication	All experiments were repeated at least twice as successful, independent experiments. Details as to the exact number of replicates are included in the Figure Legends.
Randomization	All mice within a litter were used for immune-phenotyping experiments. Both male and female age-matched littermate controls were used for experiments. No treatments or interventions requiring allocation to an experimental or control group were performed.
Blinding	Investigators were not blinded. Mice of different genotypes were treated equally and housed together.

Reporting for specific materials, systems and methods

We require information from authors about some types of materials, experimental systems and methods used in many studies. Here, indicate whether each material, system or method listed is relevant to your study. If you are not sure if a list item applies to your research, read the appropriate section before selecting a response.

Materials & experimental systems

n/a	Involved in the study
☐	☒ Antibodies
☒	☐ Eukaryotic cell lines
☒	☐ Palaeontology and archaeology
☐	☒ Animals and other organisms
☒	☐ Clinical data
☒	☐ Dual use research of concern

Methods

n/a	Involved in the study
☒	☐ ChIP-seq
☐	☒ Flow cytometry
☒	☐ MRI-based neuroimaging

Antibodies

Antibodies used

The following monoclonal fluorophore-conjugated antibodies were used in this study:

B220 - Brilliant UltraViolet 737 - RA3-6B2 - BD Biosciences - 612838 - 1/600
c-Kit - Brilliant UltraViolet 395 - 2B8 - BioLegend - 564011 - 1/800
CCR6 - Brilliant Violet 785 - 29-2L17 - BioLegend - 129823 - 1/300
CCR6 - Alexa Fluor 647 - 140706 - BD Biosciences - 557976 - 1/200
CCR6 - PE-Fluor810 - 29-2L17 - BioLegend - 129833 - 1/100
CCR7 - Alexa Fluor 488 - 4B12 - BioLegend - 120110 - 1/100
CD11b - Brilliant UltraViolet 395 - M1/70 - BD Biosciences - 563553 - 1/1000
CD11b - Brilliant Violet 785 - M1/70 - BioLegend - 101243 - 1/400
CD11c - APC-Cy7 - N418 - BioLegend - 117323 - 1/200
CD11c - Brilliant UltraViolet 563 - N418 - BD Biosciences - 749040 - 1/400
CD19 - Brilliant UltraViolet 737 - 1D3 - BD Biosciences - 612781 - 1/800
CD19 - Biotinylated - 6D5 - BioLegend - 115504 - 1/200
CD19 - Brilliant Violet 421 - 6D5 - BioLegend - 115549 - 1/400
CD19 - FITC - 1D3 - BD Biosciences - 557398 - 1/500
CD27 - PE-Cy7 - LG.7F9 - ThermoFisher - 25-0271-80 - 1/100
CD27 - FITC - LG.7F9 - ThermoFisher - 11-0271-82 - 1/1000
CD31 - FITC - MEC13.3 - BD Biosciences - 553372 - 1/200
CD3e - Biotinylated - 145-2c11 - BioLegend - 100304 - 1/200
CD45 - Brilliant Violet 570 - 30-F11 - BioLegend - 103136 - 1/600
CD45 - Brilliant Violet 510 - 30-F11 - BioLegend - 103138 - 1/2000
CD45.1 - Brilliant UltraViolet 615 - A20 - BD Biosciences - 751467 - 1/100
CD45.2 - Violet450 - 104 - Tonbo Bioscience (Fisher Scientific) - 50-105-5147 - 1/400
CD64 - PE-Cy7 - X-54-5/7.1 - BioLegend - 139314 - 1/200
CD88 - Brilliant Violet 421 - 20/70 - BD Biosciences - 743769 - 1/400
CD88 - Biotinylated - 20/70 - BioLegend - 135811 - 1/200
CD88 - PerCP-Cy5.5 - 20/70 - BioLegend - 135813 - 1/100
CD90.2 - Biotinylated - 30-H12 - BioLegend - 105304 - 1/200
CD90.2 - Brilliant Violet 786 - 53-2.1 - BD Biosciences - 564365 - 1/800
CD90.2 - Brilliant UltraViolet 496 - 53-2.1 - BD Biosciences - 741046 - 1/400
CSF1-R - Brilliant Violet 711 - AFS98 - BioLegend - 135515 - 1/100
CXCR6 - PE - SA051D1 - BioLegend - 151104 - 1/200
CXCR6 - Alexa Fluor 647 - SA051D2 - BioLegend - 151115 - 1/200
CXCR6 - APC-Fire750 - SA051D1 - BioLegend - 151129 - 1/200
EpCAM - Brilliant Violet 605 - G8.8 - BioLegend - 118227 - 1/200
EpCAM - Brilliant Violet 650 - G8.8 - BioLegend - 118241 - 1/200
EpCAM - PE-Cy7 - G8.8 - BioLegend - 118216 - 1/1000
FceR1a - Biotinylated - MAR-1 - ThermoFisher - 13-5898-82 - 1/100
FLT3 - PE-Cy7 - A2F10.1 - BD Biosciences - 567594 - 1/600
FLT3 - PE-CF594 - A2F10.1 - BD Biosciences - 562537 - 1/200
ICAM-1 - PerCP-Cy5.5 - YN1/1.7.4 - BioLegend - 116124 - 1/200
IL1R2 - PE - 4E 2 - BD Biosciences - 554450 - 1/200
IL7R - APC - A7R34 - BioLegend - 135012 - 1/200
IL7R - Brilliant Violet 785 - A7R34 - BioLegend - 135037 - 1/200
IL7R - PE - A7R34 - Tonbo Bioscience (Fisher Scientific) - 50-1271-U100 - 1/200
IL7R - Brilliant UltraViolet 737 - SB/199 - BD Biosciences - 564399 - 1/200
KLRG1 - FITC - 2F1 - ThermoFisher - 11-5893-82 - 1/200
Ly6C - eFluor 450 - RB6-8C5 - ThermoFisher - 48-5931-80 - 1/200
Ly6C - Brilliant Violet 711 - HK1.4 - BioLegend - 128037 - 1/800
Ly6D - eFluor 450 - 49-H4 - Thermo - 48-5974-80 - 1/1600
Ly6D - FITC - 49-H4 - BioLegend - 138605 - 1/3000
Ly6G - Biotinylated - 1A8 - BioLegend - 127604 - 1/200
Ly6G - APC-Fire 810 - 1A8 - BioLegend - 127669 - 1/600
MHC Class II (I-A/I-E) - PerCP-Cy5.5 - M5/114.15.2 - Tonbo Bioscience (Fisher Scientific) - 65-5321-U100 - 1/400
MHC Class II (I-A/I-E) - Alexa Fluor 700 - M5/114.15.2 - BioLegend - 107622 - 1/1300
MHC Class II (I-A/I-E) - Brilliant Violet 480 - M5/114.15.2 - BD Biosciences - 566086 - 1/800
MHC Class II (I-A/I-E) - Brilliant UltraViolet 563 - M5/114.15.2 - BD Biosciences - 748846 - 1/1500
NCAM-1 - Brilliant UltraViolet 661 - 809220 - BD Biosciences - 750021 - 1/100

NCAM-1 - Brilliant Violet 480 - 809220 (RUO) - BD Biosciences - 748095 - 1/100
Neuropilin-1 - Brilliant Violet 421 - 3 E 12 - BioLegend - 145209 - 1/400
NK1.1 - eFluor 450 - PK136 - ThermoFisher - 48-5941-82 - 1/400
NK1.1 - Biotinylated - PK136 - BioLegend - 108704 - 1/200
NK1.1 - Brilliant Violet 510 - PK136 - BioLegend - 108738 - 1/300
NKp46 - Brilliant Violet 650 - 2911.4 - BioLegend - 137635 - 1/100
PD1 - Brilliant Violet 785 - 29F.1A12 - BioLegend - 135225 - 1/400
Podoplanin - PE-Cy7 - 8.1.1 - BioLegend - 127412 - 1/400
RORgt - PE-C594 - Q31-378 - BD Biosciences - 562684 - 1/400
SiglecF - Brilliant Violet 421 - E50-2440 - BD Biosciences - 562681 - 1/400
SiglecF - PE-Cy7 - S17007L - BioLegend - 155527 - 1/500
SiglecF - PerCP-Fire 806 - S17007L - BioLegend - 155535 - 1/800
SiglecH - Percp-Cy5.5 - 551 - BioLegend - 129614 - 1/200
SiglecH - Alexa Fluor 647 - 551 - BioLegend - 129608 - 1/400
SiglecH - Brilliant UltraViolet 395 - 551 - BD Biosciences - 567814 - 1/800
SIRP-a - Brilliant Violet 605 - P84 - BD Biosciences - 740390 - 1/400
Streptavidin - Brilliant UltraViolet 737 - Streptavidin - BD Biosciences - 564293 - 1/2000
Streptavidin - Brilliant Violet 421 - Streptavidin - BioLegend - 405225 - 1/4000
TCR b - APC-Cy7 - H57-597 - BioLegend - 109220 - 1/400
TCR b - Alexa Fluor 700 - H57-597 - ThermoFisher - 56-5961-82 - 1/300
TCR b - Biotinylated - H57-597 - BioLegend - 109204 - 1/200
TCR gd - Biotinylated - UC7-13D5 - ThermoFisher - 13-5811-85 - 1/200
TCR gd - Brilliant Violet 421 - GL3 - BioLegend - 118119 - 1/400
Ter-119 - Biotinylated - TER-119 - BioLegend - 116204 - 1/200
VCAM-1 - APC - 429 (MVCAM.A) - BioLegend - 105718 - 1/200
XCR1 - Brilliant Violet 650 - ZET - BioLegend - 148220 - 1/200
XCR1 - PerCP-Cy5.5 - ZET - BioLegend - 148208 - 1/100
LIVE/DEAD Fixable Zombie NIR (Biolegend, 423105) was used at 1/2000 to exclude dead cells.
Anti-CD16/32 (Biolegend, 101320) was used at 1/100 to block binding to Fc receptors prior to cell surface staining.

Validation

All commercially available antibodies are routinely tested by the vendor. Antibody validation is provided on the suppliers 'website listed below:

B220 - Brilliant UltraViolet 737 - https://www.bdbiosciences.com/en-us/products/reagents/flow-cytometry-reagents/research-reagents/single-color-antibodies-ruo/buv737-rat-anti-mouse-cd45r-b220.612838?tab=antibody_details
c-Kit - Brilliant UltraViolet 395 - https://www.bdbiosciences.com/en-us/products/reagents/flow-cytometry-reagents/research-reagents/single-color-antibodies-ruo/buv395-rat-anti-mouse-cd117.564011?tab=product_details
CCR6 - Brilliant Violet 785 - https://sandbox.biolegend.com/en-gb/products/brilliant-violet-785-anti-mouse-cd196-ccr6-antibody-14749
CCR6 - Alexa Fluor 647 - https://www.bdbiosciences.com/en-ca/products/reagents/flow-cytometry-reagents/research-reagents/single-color-antibodies-ruo/alexa-fluor-647-rat-anti-mouse-cd196.557976?tab=product_details
CCR6 - PE-Fluor810 - https://www.biolegend.com/en-us/products/pe-fire-810-anti-mouse-cd196-ccr6-antibody-22722
CCR7 - Alexa Fluor 488 - https://www.biolegend.com/en-us/products/alexa-fluor-488-anti-mouse-cd197-ccr7-antibody-2844
CD11b - Brilliant UltraViolet 395 - https://www.bdbiosciences.com/en-us/products/reagents/flow-cytometry-reagents/research-reagents/single-color-antibodies-ruo/buv395-rat-anti-cd11b.563553?tab=product_details
CD11b - Brilliant Violet 785 - https://www.biolegend.com/en-us/products/brilliant-violet-785-anti-mouse-human-cd11b-antibody-7958
CD11c - APC-Cy7 - https://www.biolegend.com/en-us/products/apc-cyanine7-anti-mouse-cd11c-antibody-3931
CD11c - Brilliant UltraViolet 563 - https://www.bdbiosciences.com/en-us/products/reagents/flow-cytometry-reagents/research-reagents/single-color-antibodies-ruo/buv563-hamster-anti-mouse-cd11c.749040?tab=product_details
CD19 - Brilliant UltraViolet 737 - https://www.bdbiosciences.com/en-us/products/reagents/flow-cytometry-reagents/research-reagents/single-color-antibodies-ruo/buv737-rat-anti-mouse-cd19.612781?tab=product_details
CD19 - Biotinylated - https://www.biolegend.com/en-us/products/biotin-anti-mouse-cd19-antibody-1527
CD19 - Brilliant Violet 421 - https://www.biolegend.com/en-us/products/brilliant-violet-421-anti-mouse-cd19-antibody-7160
CD19 - FITC - https://www.bdbiosciences.com/en-us/products/reagents/flow-cytometry-reagents/research-reagents/single-color-antibodies-ruo/fitc-rat-anti-mouse-cd19.557398?tab=product_details
CD27 - PE-Cy7 - https://www.thermofisher.com/antibody/product/CD27-Antibody-clone-LG-7F9-Monoclonal/25-0271-82
CD27 - FITC - https://www.thermofisher.com/antibody/product/CD27-Antibody-clone-LG-7F9-Monoclonal/11-0271-82
CD31 - FITC - https://www.bdbiosciences.com/en-us/products/reagents/flow-cytometry-reagents/research-reagents/single-color-antibodies-ruo/fitc-rat-anti-mouse-cd31.553372?tab=product_details
CD3e - Biotinylated - https://www.biolegend.com/en-us/products/biotin-anti-mouse-cd3epsilon-antibody-22
CD45 - Brilliant Violet 570 - https://www.biolegend.com/en-us/products/brilliant-violet-570-anti-mouse-cd45-antibody-7452
CD45 - Brilliant Violet 510 - https://www.biolegend.com/en-us/products/brilliant-violet-510-anti-mouse-cd45-antibody-7995
CD45.1 - Brilliant UltraViolet 615 - https://www.bdbiosciences.com/en-us/products/reagents/flow-cytometry-reagents/research-reagents/single-color-antibodies-ruo/buv615-mouse-anti-mouse-cd45-1.751467?tab=product_details
CD45.2 - Violet450 - https://www.fishersci.com/shop/products/violet-450-ms-cd45-2-104-100ug/501055147?searchHijack=true&searchTerm=50-105-5147&searchType=RAPID&matchedCatNo=50-105-5147
CD64 - PE-Cy7 - https://www.biolegend.com/en-us/products/pe-cyanine7-anti-mouse-cd64-fcgammari-antibody-10062
CD88 - Brilliant Violet 421 - https://www.bdbiosciences.com/en-us/products/reagents/flow-cytometry-reagents/research-reagents/single-color-antibodies-ruo/bv421-rat-anti-mouse-cd88.743769?tab=product_details
CD88 - Biotinylated - https://www.biolegend.com/en-us/products/biotin-anti-mouse-cd88-c5ar-antibody-7785
CD88 - PerCP-Cy5.5 - https://www.biolegend.com/en-us/products/percp-cyanine5-5-anti-mouse-cd88-c5ar-antibody-16366
CD90.2 - Biotinylated - https://www.biolegend.com/en-us/products/biotin-anti-mouse-cd90-2-thy1-2-antibody-103
CD90.2 - Brilliant Violet 786 - https://www.bdbiosciences.com/en-us/products/reagents/flow-cytometry-reagents/research-reagents/single-color-antibodies-ruo/bv786-rat-anti-mouse-cd90-2.564365?tab=product_details
CD90.2 - Brilliant UltraViolet 496 - https://www.bdbiosciences.com/en-us/products/reagents/flow-cytometry-reagents/research-

reagents/single-color-antibodies-ruo/buv496-rat-anti-mouse-cd90-2.741046?tab=product_details
CSF1-R - Brilliant Violet 711 - https://www.biolegend.com/en-us/products/brilliant-violet-711-anti-mouse-cd115-csf-1r-antibody-9030
CXCR6 - PE - https://www.biolegend.com/en-us/products/pe-anti-mouse-cd186-cxcr6-antibody-12545
CXCR6 - Alexa Fluor 647 - https://www.biolegend.com/en-us/products/alexa-fluor-647-anti-mouse-cd186-cxcr6-antibody-15231
CXCR6 - APC-Fire750 - https://www.biolegend.com/en-us/products/apc-fire-750-anti-mouse-cd186-cxcr6-antibody-21764
EpCAM - Brilliant Violet 605 - https://www.biolegend.com/en-us/products/brilliant-violet-605-anti-mouse-cd326-ep-cam-antibody-9968
EpCAM - Brilliant Violet 650 - https://www.biolegend.com/en-us/products/brilliant-violet-650-anti-mouse-cd326-ep-cam-antibody-19847
EpCAM - PE-Cy7 - https://www.biolegend.com/en-us/products/pe-cyanine7-anti-mouse-cd326-ep-cam-antibody-5303
FceR1a - Biotinylated - https://www.thermofisher.com/antibody/product/FceR1-alpha-Antibody-clone-MAR-1-Monoclonal/13-5898-82
FLT3 - PE-Cy7 - https://www.bdbiosciences.com/en-us/products/reagents/flow-cytometry-reagents/research-reagents/single-color-antibodies-ruo/pe-cy7-rat-anti-mouse-cd135-flt3.567594?tab=product_details
FLT3 - PE-CF594 - https://www.bdbiosciences.com/en-us/products/reagents/flow-cytometry-reagents/research-reagents/single-color-antibodies-ruo/pe-cf594-rat-anti-mouse-cd135.562537?tab=product_details
ICAM-1 - PerCP-Cy5.5 - https://www.biolegend.com/en-us/products/percp-cyanine5-5-anti-mouse-cd54-antibody-14748
IL1R2 - PE - https://www.bdbiosciences.com/en-us/products/reagents/flow-cytometry-reagents/research-reagents/single-color-antibodies-ruo/pe-rat-anti-mouse-cd121b.554450?tab=product_details
IL7R - APC - https://www.biolegend.com/en-us/products/apc-anti-mouse-cd127-il-7ralpha-antibody-6191
IL7R - Brilliant Violet 785 - https://www.biolegend.com/en-us/products/brilliant-violet-785-anti-mouse-cd127-il-7ralpha-antibody-10803
IL7R - PE - https://www.fishersci.com/shop/products/pe-ms-cd127-a7r34-100ug/502014707?searchHijack=true&searchTerm=50-1271-U100&searchType=RAPID&matchedCatNo=50-1271-U100
IL7R - Brilliant UltraViolet 737 - https://www.bdbiosciences.com/en-us/products/reagents/flow-cytometry-reagents/research-reagents/single-color-antibodies-ruo/buv737-rat-anti-mouse-cd127.612841?tab=product_details
KLRG1 - FITC - https://www.thermofisher.com/antibody/product/KLRG1-Antibody-clone-2F1-Monoclonal/11-5893-82
Ly6C/Ly6G - eFluor 450 - https://www.thermofisher.com/antibody/product/Ly-6G-Ly-6C-Antibody-clone-RB6-8C5-Monoclonal/48-5931-80
Ly6C - Brilliant Violet 711 - https://www.biolegend.com/en-us/products/brilliant-violet-711-anti-mouse-ly-6c-antibody-8935
Ly6D - eFluor 450 - https://www.thermofisher.com/antibody/product/Ly-6D-Antibody-clone-49-H4-Monoclonal/48-5974-80
Ly6D - FITC - https://www.biolegend.com/en-us/products/fitc-anti-mouse-ly-6d-antibody-7769
Ly6G - Biotinylated - https://www.biolegend.com/en-us/products/biotin-anti-mouse-ly-6g-antibody-4772
Ly6G - APC-Fire 810 - https://www.biolegend.com/en-us/products/apc-fire-810-anti-mouse-ly-6g-antibody-21380
MHC Class II (I-A/I-E) - PerCP-Cy5.5 - https://www.fishersci.com/shop/products/percpcy5-5-ms-mhcclassii/502014823?searchHijack=true&searchTerm=65-5321-U100&searchType=RAPID&matchedCatNo=65-5321-U100
MHC Class II (I-A/I-E) - Alexa Fluor 700 - https://www.biolegend.com/en-us/products/alexa-fluor-700-anti-mouse-i-a-i-e-antibody-3413
MHC Class II (I-A/I-E) - Brilliant Violet 480 - https://www.bdbiosciences.com/en-us/products/reagents/flow-cytometry-reagents/research-reagents/single-color-antibodies-ruo/bv480-rat-anti-mouse-i-a-i-e.566086?tab=product_details
MHC Class II (I-A/I-E) - Brilliant UltraViolet 563 - https://www.bdbiosciences.com/en-us/products/reagents/flow-cytometry-reagents/research-reagents/single-color-antibodies-ruo/buv563-rat-anti-mouse-i-a-i-e.748846?tab=product_details
NCAM-1 - Brilliant UltraViolet 661 - https://www.bdbiosciences.com/en-us/products/reagents/flow-cytometry-reagents/research-reagents/single-color-antibodies-ruo/buv661-rat-anti-mouse-cd56-ncam-1.750021?tab=product_details
NCAM-1 - Brilliant Violet 480 - https://www.bdbiosciences.com/en-us/products/reagents/flow-cytometry-reagents/research-reagents/single-color-antibodies-ruo/bv480-rat-anti-mouse-cd56-ncam-1.748095?tab=product_details
Neuropilin-1 - Brilliant Violet 421 - https://www.biolegend.com/en-us/products/brilliant-violet-421-anti-mouse-cd304-neuropilin-1-antibody-8731
NK1.1 - eFluor 450 - https://www.thermofisher.com/antibody/product/NK1-1-Antibody-clone-PK136-Monoclonal/48-5941-82
NK1.1 - Biotinylated - https://www.biolegend.com/en-us/products/biotin-anti-mouse-nk-1-1-antibody-428
NK1.1 - Brilliant Violet 510 - https://www.biolegend.com/en-us/products/brilliant-violet-510-anti-mouse-nk-1-1-antibody-8615
NKp46 - Brilliant Violet 650 - https://www.biolegend.com/en-us/products/brilliant-violet-650-anti-mouse-cd335-nkp46-antibody-15729
PD1 - Brilliant Violet 785 - https://www.biolegend.com/en-us/products/brilliant-violet-785-anti-mouse-cd279-pd-1-antibody-9874
Podoplanin - PE-Cy7 - https://www.biolegend.com/en-us/products/pe-cyanine7-anti-mouse-podoplanin-antibody-6674
RORgt - PE-C594 - https://www.bdbiosciences.com/en-us/products/reagents/flow-cytometry-reagents/research-reagents/single-color-antibodies-ruo/pe-cf594-mouse-anti-mouse-ror-t.562684?tab=product_details
SiglecF - Brilliant Violet 421 - https://www.bdbiosciences.com/en-us/products/reagents/flow-cytometry-reagents/research-reagents/single-color-antibodies-ruo/bv421-rat-anti-mouse-siglec-f.562681?tab=product_details
SiglecF - PE-Cy7 - https://www.biolegend.com/en-us/products/pe-cyanine7-anti-mouse-cd170-siglec-f-antibody-20500
SiglecF - PerCP-Fire 806 - https://www.biolegend.com/en-us/products/percp-fire-806-anti-mouse-cd170-siglec-f-antibody-23384
SiglecH - Percp-Cy5.5 - https://www.biolegend.com/en-us/products/percp-cyanine5-5-anti-mouse-siglec-h-antibody-6927
SiglecH - Alexa Fluor 647 - https://www.biolegend.com/en-us/products/alexa-fluor-647-anti-mouse-siglec-h-antibody-5179
SiglecH - Brilliant UltraViolet 395 - https://www.bdbiosciences.com/en-us/products/reagents/flow-cytometry-reagents/research-reagents/single-color-antibodies-ruo/buv395-rat-anti-mouse-siglec-h.567814?tab=product_details
SIRP-a - Brilliant Violet 605 - https://www.bdbiosciences.com/en-us/products/reagents/flow-cytometry-reagents/research-reagents/single-color-antibodies-ruo/bv605-rat-anti-mouse-cd172a.740390?tab=product_details
Streptavidin - Brilliant UltraViolet 737 - https://www.bdbiosciences.com/en-us/products/reagents/flow-cytometry-reagents/research-reagents/single-color-antibodies-ruo/buv737-streptavidin.612775?tab=product_details
Streptavidin - Brilliant Violet 421 - https://www.biolegend.com/en-us/products/brilliant-violet-421-streptavidin-7297
TCR b - APC-Cy7 - https://www.biolegend.com/en-us/products/apc-cyanine7-anti-mouse-tcr-beta-chain-antibody-4137
TCR b - Alexa Fluor 700 - https://www.thermofisher.com/antibody/product/TCR-beta-Antibody-clone-H57-597-Monoclonal/56-5961-82
TCR b - Biotinylated - https://www.biolegend.com/en-us/products/biotin-anti-mouse-tcr-beta-chain-antibody-269
TCR gd - Biotinylated - https://www.thermofisher.com/antibody/product/TCR-gamma-delta-Antibody-clone-UC7-13D5-

Monoclonal/13-5811-85
TCR gd - Brilliant Violet 421 - https://www.biolegend.com/en-us/products/brilliant-violet-421-anti-mouse-tcr-gamma-delta-antibody-7249
Ter-119 - Biotinylated - https://www.biolegend.com/en-us/products/biotin-anti-mouse-ter-119-erythroid-cells-antibody-1864
VCAM-1 - APC - https://www.biolegend.com/en-us/products/apc-anti-mouse-cd106-antibody-6079
XCR1 - Brilliant Violet 650 - https://www.biolegend.com/en-us/products/brilliant-violet-650-anti-mouse-rat-xcr1-antibody-12421
XCR1 - PerCP-Cy5.5 - https://www.biolegend.com/en-us/products/percp-cyanine5-5-anti-mouse-rat-xcr1-antibody-10397
Zombie NIR - https://www.biolegend.com/en-us/products/zombie-nir-fixable-viability-kit-8657
CD16/32 - https://www.biolegend.com/en-us/products/trustain-fcx-anti-mouse-cd16-32-antibody-5683

Animals and other research organisms

Policy information about studies involving animals; ARRIVE guidelines recommended for reporting animal research, and Sex and Gender in Research

Laboratory animals	AireGreenLantern-T2A-creERT2 and Prdm16-P2A-iCreERT2-T2A-mBaoJin KI mice (referred to as Prdm16creERT2-mBaojin) were generated as described in the manuscript. RorcVenus-creERT2 (referred to as RorcVenus), Il7rcre, Spi1fl/fl, Toxfl/fl, Tcf7fl/fl, Tnfrsf11acre-GFP, Tnfrsf11afl/fl, Tnfsf11fl/fl, Twist2cre, Prdm16fl/fl R26:FlpoERT2 Flt3Frt-ITD, Siglechcre and Tcf4STOP mice have been previously described. Rorgtcre, RorgtGFP, R26lsl-tdTomato(Ai14), Cx3cr1cre, C57Bl/6 (CD45.2) and CD45.1 (PtprcK302E) mice were purchased from Jackson Laboratories. Siglecfcre mice (Cat. NO. NMKI-231128) were purchased from Shanghai Model Organisms. Mice were analyzed at 2 weeks of age unless otherwise stated.
Wild animals	No wild animals were used in this study.
Reporting on sex	Both male and females were used in the study and we did not observe any sex-specific phenotypes.
Field-collected samples	No field-collected samples were used in this study.
Ethics oversight	Generation and treatments of mice were performed under protocol 21-05-007, approved by the Sloan Kettering Institute (SKI) Institutional Animal Care and Use Committee.

Note that full information on the approval of the study protocol must also be provided in the manuscript.

Flow Cytometry

Plots

Confirm that:

☒ The axis labels state the marker and fluorochrome used (e.g. CD4-FITC).

☒ The axis scales are clearly visible. Include numbers along axes only for bottom left plot of group (a 'group' is an analysis of identical markers).

☒ All plots are contour plots with outliers or pseudocolor plots.

☒ A numerical value for number of cells or percentage (with statistics) is provided.

Methodology

Sample preparation	Sample preparation was performed as described in the methods section
Instrument	BD Aria or Cytek Aurora CS for cell sorting, Cytek Aurora for flow cytometry
Software	Flowjo v10.10.0
Cell population abundance	Frequencies of cell populations are indicated on the flow plots.
Gating strategy	A detailed gating strategy is included in the supplementary information, main or extended figures.

☒ Tick this box to confirm that a figure exemplifying the gating strategy is provided in the Supplementary Information.
