# Supplementary material for: Author Correction: Ontogeny and transcriptional regulation of Thetis cells
Source: Nature. 2026 Jun 16;655(8121):E2. doi: 10.1038/s41586-026-10770-7 (PMC13323067; doi:10.1038/s41586-026-10770-7)

nature portfolio

https://doi.org/10.1038/s41586-026-10770-7

Supplementary information

Author Correction: Ontogeny and transcriptional regulation of Thetis cells

In the format provided by the authors and unedited

Ontogeny and transcriptional regulation of Thetis cells

Yoselin A. Paucar Iza, Tyler Park, Eliyambuya Baker, Gayathri Shibu, Tilman Hoelting, Greyson Feather, Anushka Yadav, Yollanda Franco Parisotto, Zihan Zhao, Blossom Akagbosu, Marc Elosua Bayes, Logan Fisher, Lucas M. James, Jianping Ma, Benjamin D. Philpot, Behdad Afzali, Christina Leslie, Chrysothemis C. Brown

Correction to: *Nature* https://doi.org/10.1038/s41586-026-10198-z. Published online 3rd February 2026.

Figure 5i, original

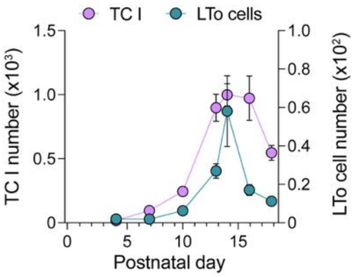

Figure 5i, corrected

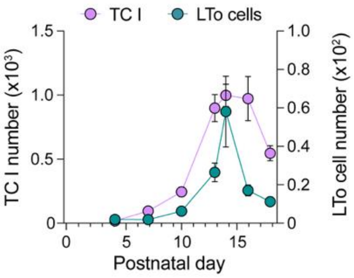

Extended Data Figure 6c
top right, original

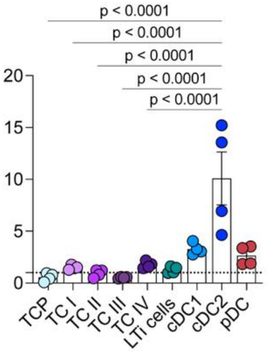

Extended Data Figure 6c
top right, corrected

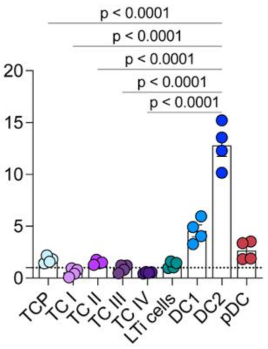

Extended Data Figure 6c
bottom middle, original

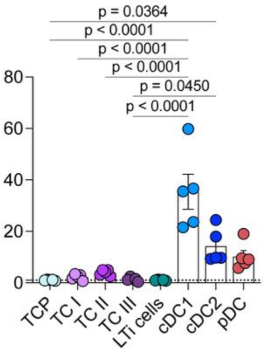

Extended Data Figure 6c
bottom middle, corrected

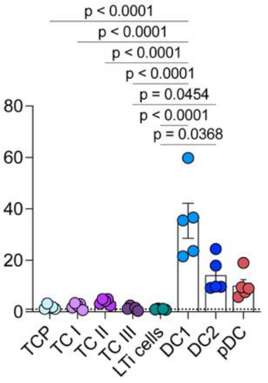

Supplement: Supplementary file 1 — Original and corrected Fig. 5i, Extended Data Fig. 6c. [file 41586_2026_10770_MOESM1_ESM.pdf]
